# ATF3 overexpression is associated with cardiac hypertrophy and electrical dysfunction accompanied by enhanced cardiac cell proliferation in zebrafish

**DOI:** 10.1038/s41598-025-33025-3

**Published:** 2025-12-26

**Authors:** Eunmi Kim, Jinho Kim, Hyun-Yi Moon, Ji Yeon Kim, Myong-Ho Jeong, Geun-Young Kim, Seung Hee Lee, Chul-Hong Kim, Jung-Woong Kim, Won-Ho Kim

**Affiliations:** 1 Division of Cardiovascular Disease Research, Department of Chronic Disease Convergence Research, National Institute of Health, 187, Osongsaengmyeong 2-Ro, Osong-Eup, Heungdeok-Gu, Cheongju, Chungcheongbuk-Do 28159 Republic of Korea; 2 Department of Life Science, Chung-Ang University, Seoul, 06974 Republic of Korea

**Keywords:** ATF3, Cardiac hypertrophy, Electrical dysfunction, Zebrafish, Cardiology, Genetics, Molecular biology, Physiology

## Abstract

Activating transcription factor 3 (ATF3) is a crucial regulator of gene expression in response to physiological stress across various tissues. Abnormal ATF3 expression is associated with cardiac dysfunction; however, the mechanisms by which it affects cardiac pathology remain unclear. In this study, we developed a cardiac-specific ATF3-expressing zebrafish line, Tg(*myl7*:*ATF3*), using a well-established vertebrate model for cardiovascular research to investigate the role of human ATF3 in the zebrafish heart. We conducted morphological assessments, immunofluorescence staining, electrocardiography, and transcriptome analysis on transgenic and wild-type zebrafish. Compared to wild-type siblings, Tg(*myl7*:*ATF3*) zebrafish exhibited significant pathophysiological changes, including cardiac hypertrophy and features resembling hypertrophic cardiomyopathy. Histological analysis revealed increased fibrotic scarring and disorganized sarcomeres. Electrocardiogram measurements indicated that ATF3 overexpression induced symptoms resembling long QT syndrome, suggesting electrical dysfunction. Transcriptome analysis demonstrated downregulation of apoptosis-related genes and upregulation of proliferation-related genes in the hearts of transgenic zebrafish. These findings suggest that ATF3 expression is associated with cardiac hypertrophy accompanied by increased proliferation of cardiac cells, including cardiomyocytes. The findings of this study provide novel insights into the role of ATF3 in cardiovascular disease progression.

**Supplementary Information:**

The online version contains supplementary material available at 10.1038/s41598-025-33025-3.

## Introduction

Activating transcription factor 3 (ATF3), a member of the ATF/cAMP response element-binding protein family, is a transcription factor that regulates gene expression. ATF3 expression is induced in response to various cellular stresses, including oxidative stress, inflammation, and DNA damage^1^. It has been implicated in both adaptive and maladaptive responses to stressors in cardiovascular health. However, the role of ATF3 in the heart remains controversial, particularly in mouse studies. Mice with cardiac-specific ATF3 overexpression exhibit enlarged atria, dilated right ventricles, increased expression of hypertrophy-associated genes, fibrosis, and myocyte degeneration^2^. Additionally, ATF3 overexpression in adult mice causes ventricular hypertrophy, cardiac dysfunction, and fibrosis^3^. Notably, wild-type (WT) mice show increased macrophage infiltration when subjected to phenylephrine infusion, whereas ATF3 knockout (KO) mice exhibit no change in macrophage numbers. This finding suggests that ATF3 expression adversely affects the heart and plays a major role in macrophage infiltration^4^. Conversely, a study comparing WT mice with aortic banding-induced cardiac hypertrophy to ATF3 KO mice observed increased cardiac hypertrophy, dysfunction, and fibrosis in the ATF3 KO group^5^. Another study using mice with cardiac-specific ATF3 KO reported increased cardiac fibrosis, inflammation, and hypertrophic marker expression in ATF3 KO mice compared to WT mice when subjected to a high-fat diet^6^, suggesting a protective effect of ATF3 in cardiac hypertrophy. These findings highlight the intricate effect of ATF3 on both cardiac physiology and pathology. However, the reported pleiotropic effects of ATF3 have yielded conflicting results, highlighting the need for further investigation. To address this controversy, the effects of ATF3 on the heart were investigated using a zebrafish model.

The zebrafish is a widely used vertebrate model for cardiovascular research, owing to its optical transparency, genetic accessibility, and conserved cardiac physiology. These characteristics enable direct in vivo visualization and functional analysis of cardiac phenotypes at single-cell resolution. This advantage makes the zebrafish particularly suitable for investigating stress-responsive transcription factors such as ATF3, whose cardiac functions and in vivo effects on myocardial remodeling remain poorly understood. Few studies have investigated ATF3 in zebrafish, and the existing literature is limited. Previous studies have primarily focused on the role of ATF3 in neural regeneration, highlighting its involvement in regeneration after optic nerve, dorsal root ganglion neuron, and spinal cord injuries^7–11^. Zebrafish larvae lacking ATF3 (*atf3* KO) exhibit dilated atrial cardiomyopathy. ATF3 has also been implicated in the differentiation of both cardiac and hematopoietic progenitors during development^12^. However, the functional implications of ATF3 gain-of-function in the zebrafish heart remain unclear, and elucidating its pleiotropic functions in mouse models poses a considerable challenge. Therefore, in this study, we generated a zebrafish model with cardiac-specific ATF3 expression, designated as Tg(*myl7:ATF3*), to investigate the role of human ATF3 in the hearts of zebrafish. We hypothesized that ATF3 expression in cardiomyocytes is associated with hypertrophy and ion channel dysfunction within these cells, resulting in abnormal cardiac structure and function in zebrafish hearts.

## Results

### Generation of a zebrafish model to investigate the conserved function of human ATF3

**Fig. 1:**
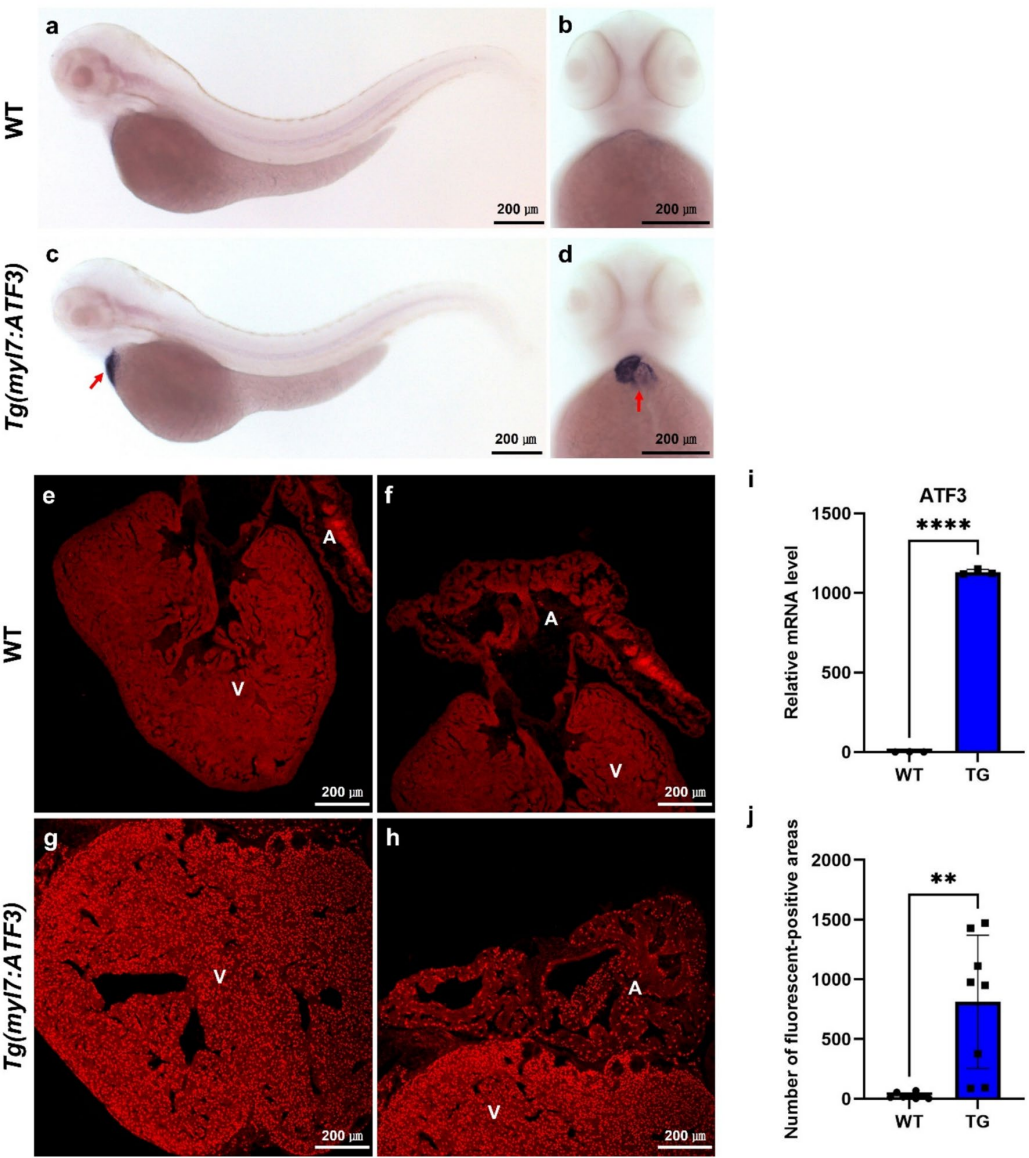
Confirmation of cardiac-specific expression of human ATF3 in Tg(*myl7:ATF3*) zebrafish. (**a**–**d**) In situ RNA hybridization for human ATF3 mRNA transcript at 3 dpf in Tg(*myl7:ATF3*) zebrafish. (**a**, **b**) WT and (**c**, **d**) Tg(*myl7:ATF3*) larvae were analyzed (n = 15 zebrafish per group). (**a**, **c**) Lateral and (**b**, **d**) ventral views show heart-specific expression of human ATF3 in Tg(*myl7:ATF3*) larvae, with red arrows indicating the heart region in (**c**, **d**). (**e**–**h**) Human ATF3 antibody staining in adult zebrafish hearts (12 mpf) (n = 3 hearts per group). (**e**, **f**) WT and (**g**, **h**) Tg(*myl7:ATF3*) hearts, with Tg showing ATF3 expression specifically in the ventricle and atrium. (**i**) Quantitative PCR analysis of ATF3 mRNA expression in hearts at 4 mpf using cardiac cDNA (n = 3 hearts per group). (**j**) Quantification of ATF3 immunofluorescence signal shown in (**e**–**h**), indicating the number of ATF3-positive positions per × 200 field (n = 3 hearts per group, with 2–3 ventricular regions analyzed per heart). Data are presented as the mean ± SD and were analyzed using an unpaired two-tailed t-test. **P < 0.01, ****P < 0.0001. *dpf* days post-fertilization, *hpf* hours post-fertilization, *mpf* months post-fertilization, *ATF3* activating transcription factor 3, *WT* wild-type, *A* atrium, *V* ventricle.

The homology of the zebrafish *atf3* ortholog was compared with mammalian ATF3 sequences to evaluate the suitability of using zebrafish as a model system for functional ATF3 studies. The amino acid sequences of ATF3 orthologs from *Homo sapiens* (ENSP00000344352), *Mus musculus* (ENSMUSP00000027941), and *Danio rerio* (ENSDARP00000027550) were aligned. The alignment revealed Percentate ID values of 95% between human and mouse and 71% between human and zebrafish. However, the amino acid sequence homology within the basic leucine zipper domain, the most important domain for ATF3 functional regulation, was 88.89% between human and zebrafish ATF3, demonstrating strong conservation (Supplementary Fig. S1). Therefore, zebrafish were used as a genetic vertebrate model to study human ATF3. Transgenic zebrafish expressing human ATF3 specifically in cardiac tissue were initially established to examine the role of the human *ATF3* gene in zebrafish cardiomyocytes. The Tg(*myl7:ATF3*) line expressed human *ATF3* under the control of the cardiomyocyte-specific promoter *myl7* (also known as *cmlc2*; Fig. 1). Whole-mount in situ RNA hybridization in zebrafish larvae revealed heart-specific expression of human ATF3 mRNA transcripts in Tg(*myl7:ATF3*) zebrafish at 3 days post-fertilization (dpf; Fig. 1a–d). Furthermore, human ATF3 was continuously expressed in the hearts of adult Tg(*myl7:ATF3*) zebrafish (Fig. 1e–j). These findings suggest that Tg(*myl7:ATF3*) zebrafish serve as a valuable model for investigating the gain-of-function effects associated with human ATF3 in the heart.

We examined whether endogenous *atf3* is induced under cardiac stress conditions to validate the physiological relevance of ATF3 expression. Zebrafish larvae at 3 dpf were treated with 20 µM terfenadine, a well-established cardiotoxic compound that induces heart failure-like symptoms and pericardial edema in zebrafish^13^, for 24 h. Larvae treated with terfenadine exhibited visible pericardial edema and a significant increase in *atf3* mRNA expression compared to DMSO controls (Supplementary Fig. S2). These results indicate that *atf3* is stress-inducible in zebrafish hearts, supporting the physiological relevance of human ATF3 overexpression in the transgenic model.

### Ectopic expression of ATF3 is associated with hypertrophic cardiomyopathy-like phenotypes in zebrafish

**Fig. 2:**
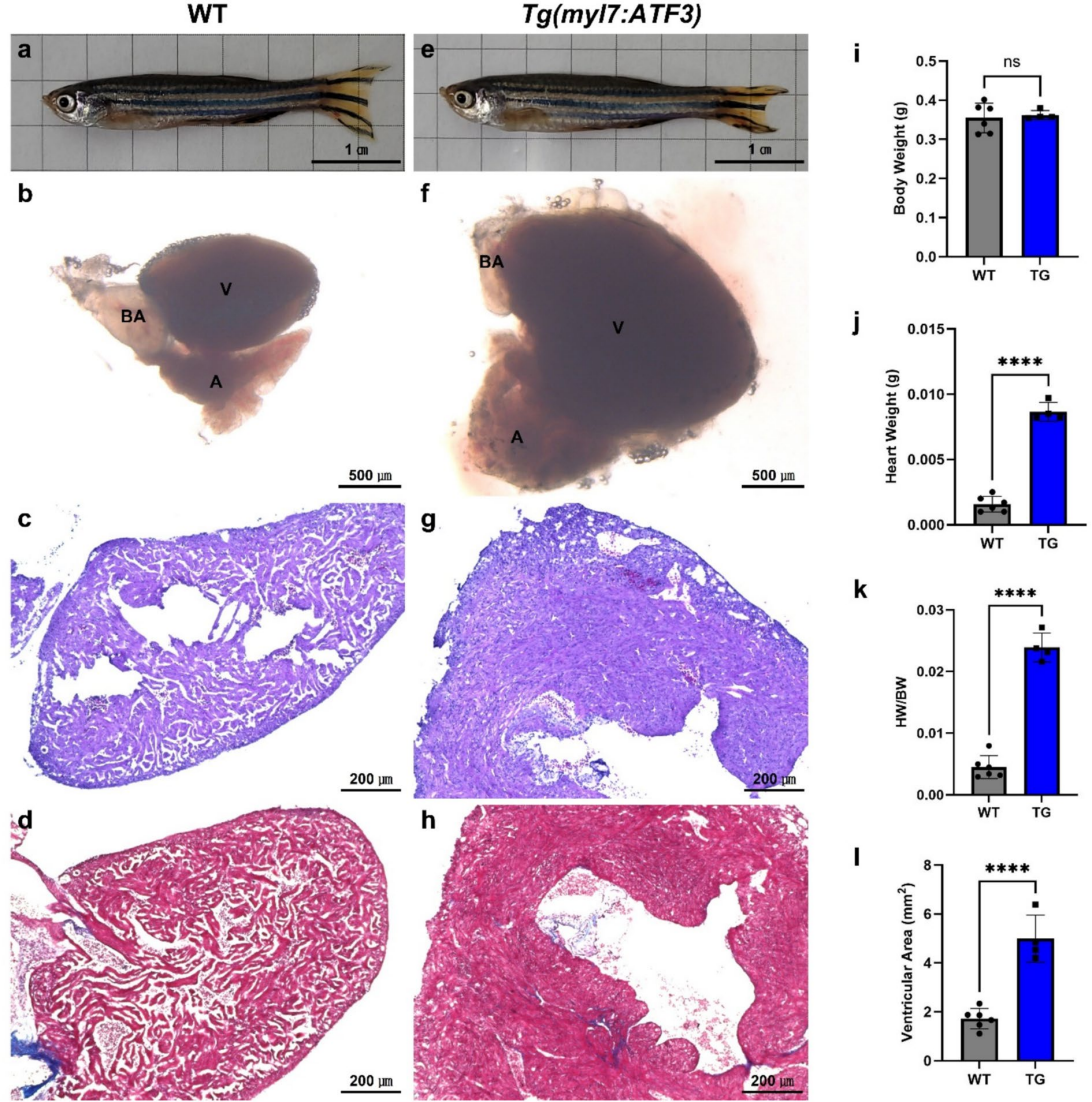
Cardiac hypertrophy in Tg(*myl7:ATF3*) adult zebrafish. (**a**–**d**) WT and **(e–h)** Tg(*myl7:ATF3*) zebrafish at 12 mpf (n = 6 WT, n = 4 TG male zebrafish). (**a**, **e**) Body length measurement and (**b**, **f**) heart size comparison. (**c**, **g**) Hematoxylin and eosin staining of WT and Tg(*myl7:ATF3*) zebrafish hearts, respectively. WT hearts display normal trabecular myocardium, whereas Tg(*myl7:ATF3*) hearts exhibit an HCM-like phenotype with thickened trabecular myocardium. (**d**, **h**) Trichrome staining of WT and Tg(*myl7:ATF3*) hearts, showing fibrosis in Tg(*myl7:ATF3*) zebrafish, consistent with HCM-like features. (**i**–**l**) Quantification of (**i**) body weight, (**j**) heart weight, (**k**) heart weight/body weight ratio, and (l) ventricular area. Quantifications were performed using the same individuals shown in the representative images. Data represent male zebrafish, and similar trends were observed in females. Although body length and body weight do not differ between WT and Tg(*myl7:ATF3*), (**j**) heart weight, (**k**) heart weight/body weight ratio, and (**l**) ventricular area are significantly increased in Tg(*myl7:ATF3*). Data are presented as the mean ± SD and were analyzed using an unpaired two-tailed t-test. *ns* not significant; ****P < 0.0001. *HCM* hypertrophic cardiomyopathy, *A* atrium, *V* ventricle, *BA* bulbus arteriosus.

The hearts of adult Tg(*myl7:ATF3*) zebrafish were examined to investigate the role of ATF3 in cardiac function. No significant differences in body length or weight were observed between the control group and Tg(*myl7:ATF3*) adult zebrafish (Fig. 2a,e,i). However, Tg(*myl7:ATF3*) adult zebrafish exhibited an enlarged heart (Fig. 2b,f,j,k). Quantitative measurement of ventricular area further validated this observation, revealing an approximately 2.5- to threefold enlargement of ventricular size in Tg(*myl7:ATF3*) zebrafish compared with WT zebrafish (Fig. 2l), with no apparent sex-dependent differences (Supplementary Table 1). The hearts of Tg(*myl7:ATF3*) zebrafish displayed a more compact and thickened ventricular wall compared to control WT zebrafish, resembling phenotypes associated with hypertrophic cardiomyopathy (HCM; Fig. 2c,g). Furthermore, trichrome staining revealed fibrotic lesions in the hearts of Tg*(myl7:ATF3)* zebrafish, whereas no fibrosis was observed in WT controls (Fig. 2d,h).

**Fig. 3:**
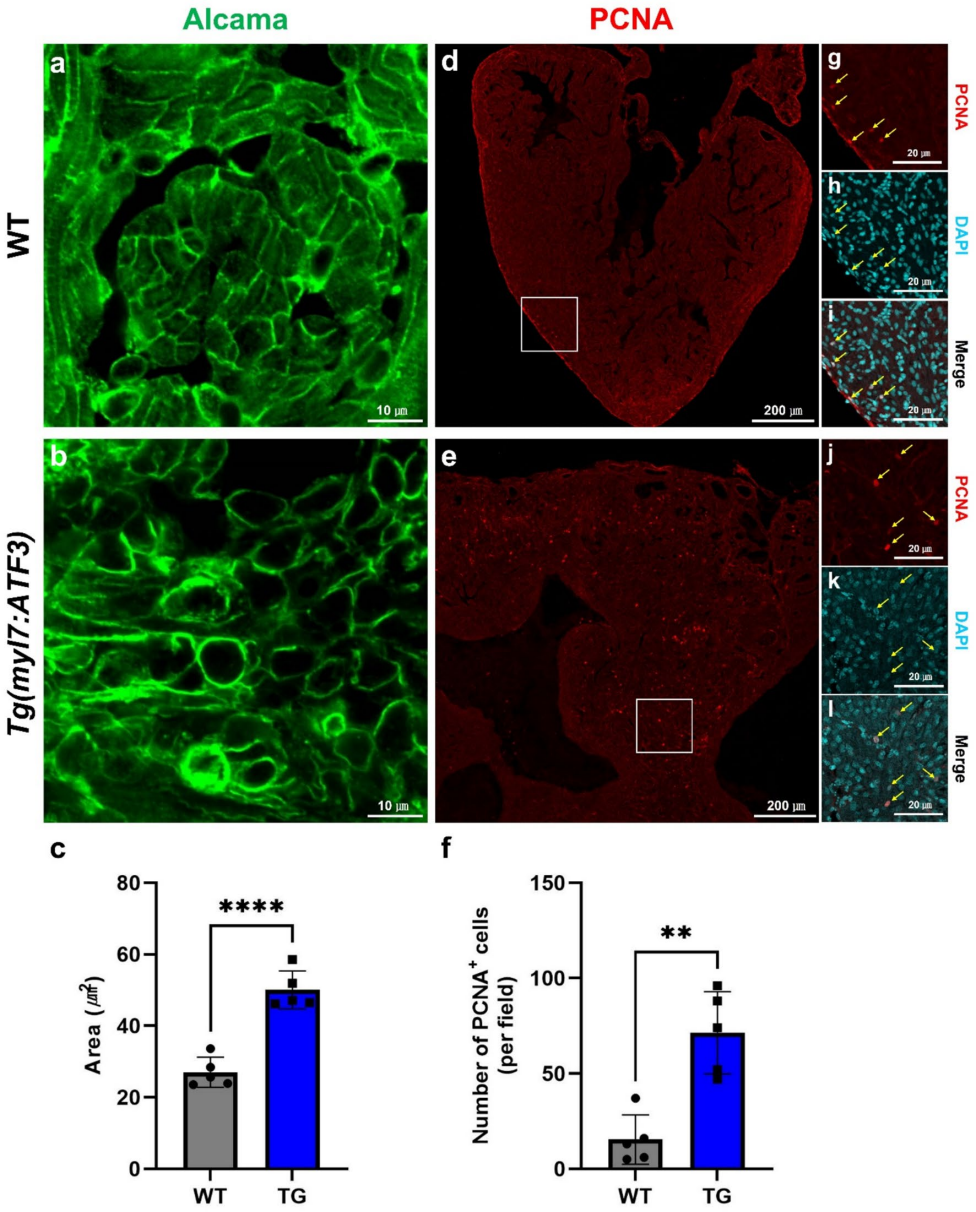
Cardiomyocyte hypertrophy and increased cell proliferation in Tg(*myl7:ATF3*) zebrafish hearts. (**a**, **b**) Immunofluorescence staining of cardiomyocytes using the Alcama antibody in (**a**) WT and (**b**) Tg(*myl7:ATF3*) hearts at 4 mpf. (**c**) Quantification of cardiomyocyte cell area reveals a significant increase in Tg(*myl7:ATF3*) zebrafish compared with WT zebrafish (n = 5 hearts per group). (**d**, **e**) Low-magnification PCNA immunofluorescence images in (**d**) WT and (**e**) Tg(*myl7:ATF3*) hearts at 12 mpf were obtained to evaluate cell proliferation. (**f**) Quantification of PCNA^+^ cells was performed by counting all PCNA^+^ nuclei within the ventricular regions captured the low-magnification images shown in (d, e), indicating significantly increased proliferation in Tg(*myl7:ATF3*) zebrafish (n = 5 hearts per group). Data are presented as the mean ± SD and were analyzed using an unpaired two-tailed t-test. **P < 0.01, ****P < 0.0001. (**g**–**l**) High-magnification views of selected regions from (**d**, **e**), showing co-staining of PCNA (red) with DAPI (cyan) to confirm nuclear localization of proliferating cells. PCNA-positive signals are confined within DAPI-labeled nuclei, validating the specificity of the proliferation assay. *PCNA* proliferating cell nuclear antigen.

Immunostaining was performed to analyze ATF3 expression and its possible link to cardiac dysfunction to investigate the association between ATF3 expression in myocardial cells and the HCM-like phenotype. Alcama was used to label the cardiomyocyte surface, facilitating the assessment of cardiomyocyte size, while proliferating cell nuclear antigen (PCNA) served as a marker to confirm cell proliferation (Fig. 3a–l). Compared to WT zebrafish hearts, those of Tg(*myl7:ATF3*) zebrafish exhibited a significant increase in cardiomyocyte size and markedly elevated cell proliferation. Phospho-histone H3 (PH3) immunostaining was performed in *Tg(myl7:ATF3);myl7:GFP* zebrafish to further characterize the proliferating population. Although some PH3-positive cells co-localized with GFP-positive cardiomyocytes, others did not, indicating that both cardiomyocytes and non-cardiomyocytes contribute to the proliferative response (Supplementary Fig. S3). These findings suggest that aberrant ATF3 upregulation is associated with cardiomyocyte hypertrophy, contributing to the development of an HCM-like phenotype.

### Analysis of cardiac structure and function in Tg(myl7:ATF3) zebrafish

The cardiac structure of zebrafish was examined using Tg(*fli1a:GFP*) zebrafish and Laminin antibody, which specifically label the endocardium and cortical myocardium^14,15^. Compared with control Tg(*fli1a:GFP*) zebrafish hearts, the structure of the *fli1a:GFP*^+^ endocardium was significantly disrupted in double-transgenic Tg(*myl7:ATF3*);Tg(*fli1a:GFP*) zebrafish hearts (Supplementary Fig. S4). Additionally, the thickness of the Laminin^+^ cortical myocardium was significantly reduced. Quantification showed that the Laminin⁺ cortical myocardial layer was reduced by approximately 72% in Tg(*myl7:ATF3*) zebrafish. In contrast to the previously observed thickening of the trabecular myocardium, a thinning of the cortical myocardium was noted in Tg(*myl7:ATF3*) zebrafish hearts (Supplementary Fig. S4). The combination of endocardial disruption, cortical thinning, and trabecular expansion indicates a global impairment of ventricular wall organization rather than a layer-specific remodeling event. Furthermore, transmission electron microscopy (TEM) analysis revealed disorganized myofibril sarcomere structures within cardiomyocytes of Tg(*myl7:ATF3*) zebrafish hearts compared with the WT controls (Supplementary Fig. S4). These findings suggest that aberrant ATF3 expression affects cardiomyocyte growth and structure, disrupting the overall architecture of the heart. Furthermore, ATF3 expression increased macrophage infiltration (Supplementary Fig. S4). Collectively, these results suggest that Tg(*myl7:ATF3*) adult zebrafish exhibit an enlarged heart with increased trabecular cardiomyocyte density, myofibril disarray, and fibrosis resembling HCM.

**Fig. 4:**
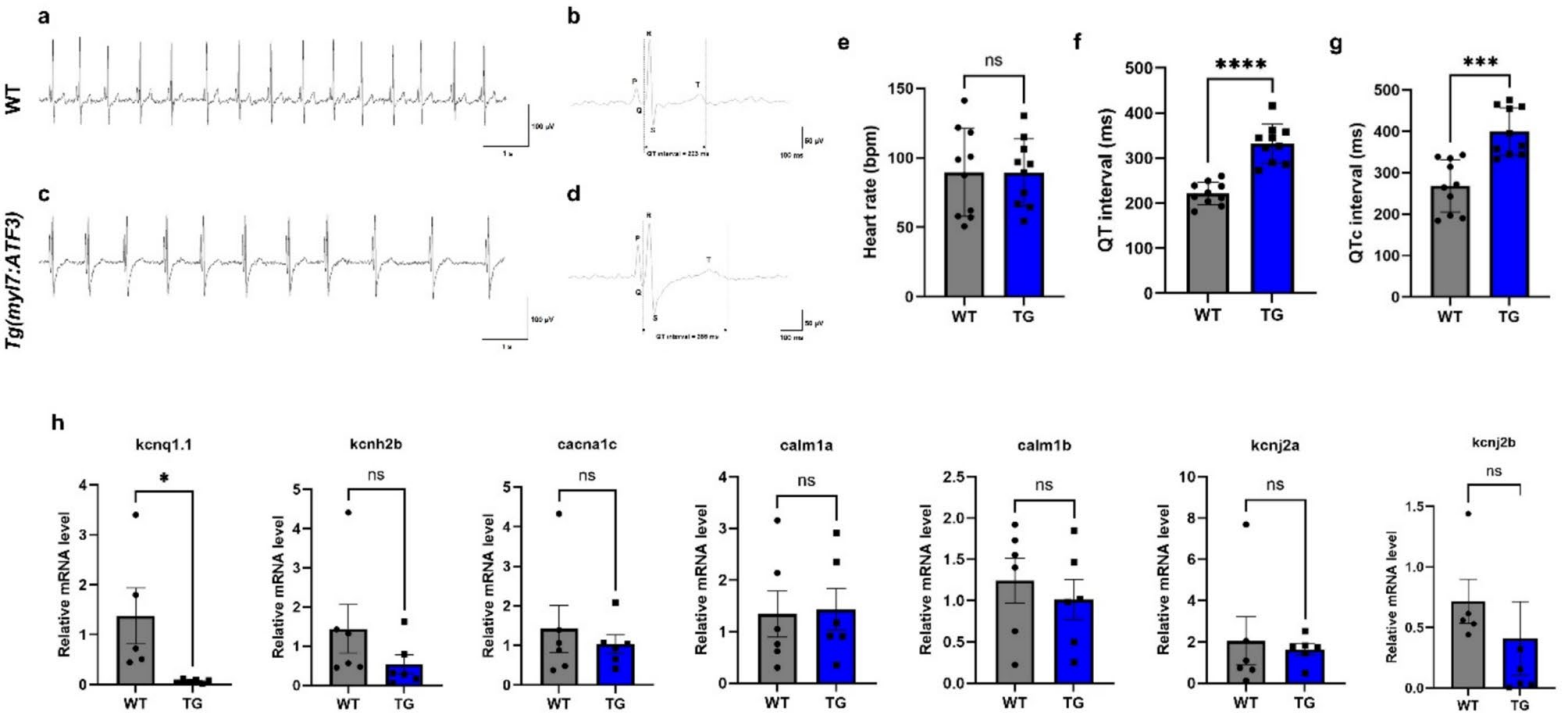
Prolonged QT and QTc intervals in Tg(*myl7:ATF3*) zebrafish. (**a**–**d**) Representative ECG traces illustrate peak intervals in (**a**, **b**) WT and (**c**, **d**) Tg(*myl7:ATF3*) zebrafish. An increased QT interval is observed in Tg(*myl7:ATF3*) compared with WT. (**e**–**g**) ECG analysis in WT and Tg(*myl7:ATF3*) zebrafish at 4 mpf (n = 10 zebrafish per group). (**e**) Heart rate (bpm) does not significantly differ between the groups. (**f**, **g**) QT interval and QTc are significantly prolonged in Tg(*myl7:ATF3*) zebrafish compared with WT zebrafish. (**h**) Quantitative PCR analysis of LQTS-related genes (*kcnq1.1, kcnh2b, cacna1c, calm1a, calm1b, kcnj2a,* and *kcnj2b*) in 4 mpf zebrafish hearts (n = 6 hearts per group). Data are presented as the mean ± SD for ECG analysis (**e**–**g**) and as the mean ± SEM for qPCR data (**h**). Statistical significance was determined using an unpaired two-tailed t-test. *ns* not significant; *P < 0.05, ***P < 0.001; ****P < 0.0001. *ECG* electrocardiogram, *QTc* corrected QT interval, *LQTS* long QT syndrome.

After analyzing the cardiac structure in response to elevated ATF3 expression, cardiac function was assessed by performing an electrocardiogram (ECG) on Tg(*myl7:ATF3*) zebrafish (Fig. 4a–d). Compared with WT zebrafish hearts, no significant difference in heart rate was observed (Fig. 4e). However, a notable prolongation of the QT interval was detected, resembling the phenotype observed in long QT syndrome (LQTS; Fig. 4f–g). These findings suggest an association between the HCM-like phenotype and abnormal cardiac functions associated with aberrant ATF3 expression. To further examine whether ATF3 overexpression affects ion channel genes related to LQTS, quantitative PCR was performed on seven representative genes (*kcnq1.1, kcnh2b, cacna1c, calm1a, calm1b, kcnj2a,* and *kcnj2b*) using cardiac cDNA from 4-month post-fertilization (mpf) zebrafish hearts (Fig. 4h). *kcnq1.1* expression was significantly decreased in Tg(*myl7:ATF3*) zebrafish compared to WT controls, whereas the other genes showed no significant differences. These results suggest that reduced *kcnq1.1* expression may contribute to the prolonged QT interval observed in Tg(*myl7:ATF3*) zebrafish.

### Profiling gene expression in Tg(myl7:ATF3) zebrafish cardiomyocytes

**Fig. 5:**
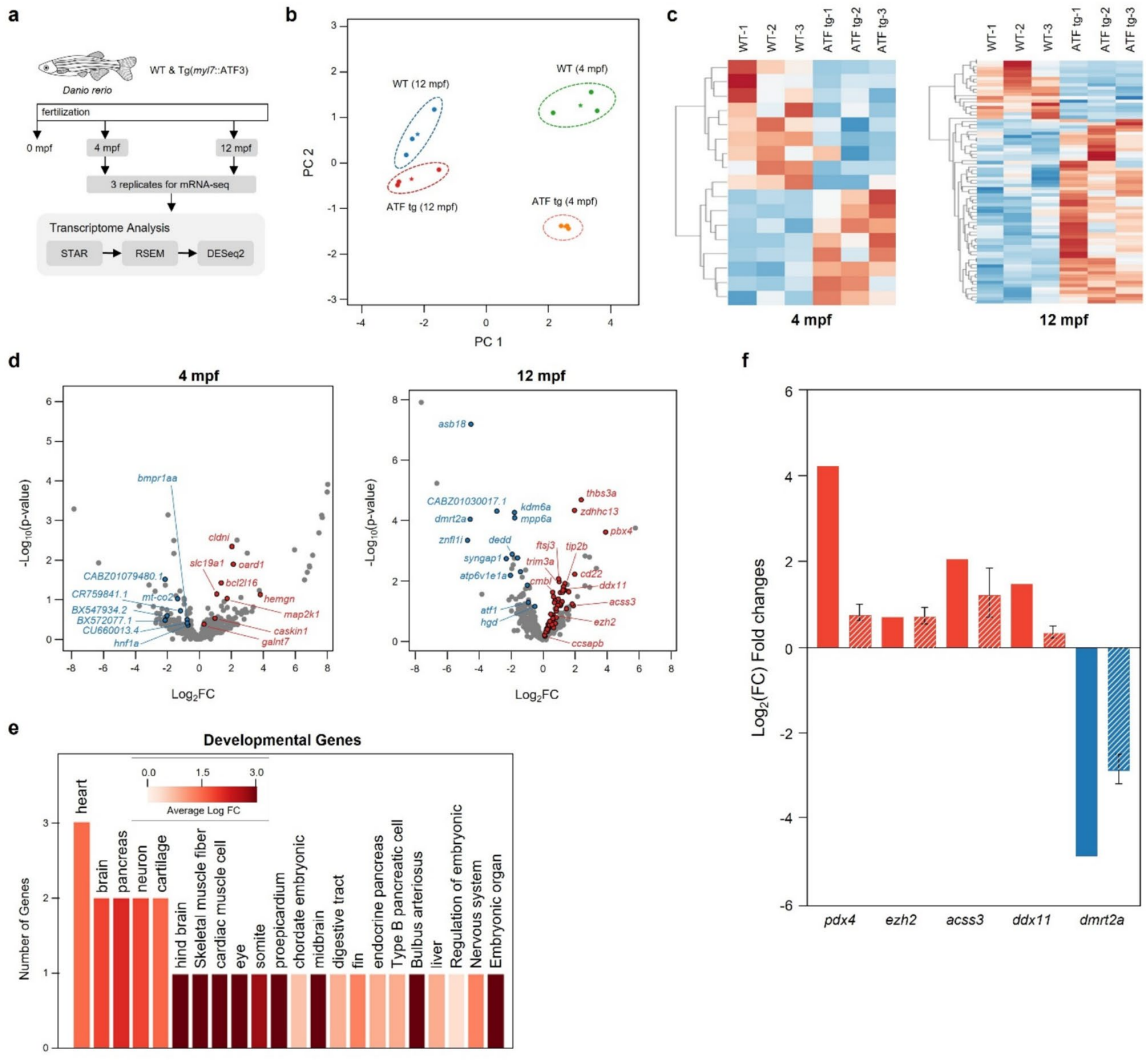
Analysis of transcriptome dynamics in Tg(*myl7:ATF3*) zebrafish. (**a**) Overview of the RNA-seq experimental design and transcriptome analysis pipeline for Tg(*myl7:ATF3*) zebrafish. (**b**) Principal component analysis plot of RNA-seq data from 4 and 12 mpf of Tg(*myl7:ATF3*) zebrafish. Each circle represents an individual sample, whereas stars indicate the average position of each group (n = 3 hearts per group). (**c**) Clustered heatmap of DEGs in 4 mpf (left) and 12 mpf (right) groups. Expression values are represented as Z-scores. (**d**) Volcano plot illustrates DEG patterns in the 4 mpf (left) and 12 mpf (right) groups. Significantly upregulated genes are highlighted in red, whereas downregulated genes are shown in blue. (**e**) GO analysis of upregulated genes in the 12 mpf group. The y-axis represents the number of genes associated with each developmental GO term, and the color of the bars indicates log_2_FC values. (**f**) Validation of RNA-seq-identified DEGs by qPCR. Graphs show comparative expression levels of selected DEGs identified from RNA-seq analysis and their corresponding qPCR validation in 12 mpf WT and Tg(*myl7:ATF3*) adult hearts. Solid bars represent RNA-seq expression changes, and dashed bars indicate qPCR results. *DEG* differentially expressed gene, *FPKM* fragments per kilobase million, *GO* Gene Ontology, *log*_*2*_*FC* log_2_ fold change.

Quantitative RNA sequencing was performed on heart tissue samples collected at 4 and 12 mpf to identify differentially expressed genes (DEGs) in *Tg(myl7:ATF3)* zebrafish cardiomyocytes (Fig. 5a). Principal component analysis (PCA) was conducted to identify the major sources of variance among groups and to confirm the reproducibility of biological replicates (Fig. 5b). For the PCA plot, genes were selected based on the following criteria: a coefficient of variation less than 80% across all groups and total fragments per kilobase million (FPKM) greater than 20 across all samples. The selected FPKM values were log10-transformed for analysis. A heatmap was generated to illustrate the expression patterns of DEGs across experimental conditions (Fig. 5c). DEGs were defined as genes meeting the following thresholds: total FPKM greater than 20 across all samples, an absolute log₂ fold change (log₂FC) greater than 0.4, and a coefficient of variation less than 1.0 in at least one group. Genes with extreme outlier expression values not captured by the coefficient of variation filter were manually excluded to further refine the dataset and minimize technical bias. Eight upregulated and nine downregulated genes were identified in the 4 mpf group, whereas 57 upregulated and 18 downregulated genes were detected in the 12 mpf group (Supplementary Table 2). Volcano plot analysis was performed to visualize the statistical significance and corresponding expression levels of DEGs (Fig. 5d). These genes were mapped to developmental and tissue-specific categories using Gene Ontology (GO) analysis to investigate the biological relevance of the DEGs identified in the 12 mpf group (Fig. 5e). Among the 57 upregulated genes, three were associated with the GO biological process term heart development (average log₂FC = 1.49). Two genes were mapped to each of the following GO biological process categories: brain development (log₂FC = 1.82), pancreas development (log₂FC = 2.07), neuron development (log₂FC = 1.82), and cartilage development (log₂FC = 1.52). These results suggest that prolonged ATF3 overexpression in cardiomyocytes induces significant transcriptional reprogramming, affecting pathways involved in tissue development and metabolic regulation. This reprogramming likely contributes to the observed cardiac remodeling and pathological changes in *Tg(myl7:ATF3)* zebrafish hearts.

### Differential expression of apoptotic, synaptic, and developmental genes in Tg(myl7:ATF3) zebrafish

Representative DEGs associated with apoptosis, synaptic function, development, and cell proliferation were selected to further elucidate the transcriptional changes induced by ATF3 overexpression (Fig. 5f, Supplementary Fig. S5). *syngap1b* (log₂FC = −2.37, P = 0.0019), *dmrt2a* (log₂FC = −4.80, P = 7.30e-4), *dedd* (log₂FC = −1.99, P = 0.0014), *buc2l* (log₂FC = −3.70, P = 0.0082), and *atp6v1e1a* (log₂FC = −2.11, P = 0.0068) exhibited significantly reduced expression, suggesting suppressed apoptotic and synaptic signaling in *Tg(myl7:ATF3)* zebrafish (Supplementary Fig. S5). The downregulation of *Syngap1b*, a pivotal regulator of synaptic plasticity, indicates potential disruptions in cellular communication. In contrast, decreased *dedd* expression aligns with reduced apoptotic activity, supporting the observed lack of notable cardiomyocyte death. Conversely, the genes associated with developmental processes and cell proliferation were significantly upregulated, including *pbx4* (log₂FC = 4.29, P = 0.00012), *thbs3a* (log₂FC = 2.66, P = 8.0e-6), and *ddx11* (log₂FC = 1.55, P = 0.014), were significantly upregulated (Supplementary Fig. S5). In contrast, *ezh2* (log₂FC = 0.77, P = 0.21) and *acss3* (log₂FC = 2.11, P = 0.057) showed a tendency toward upregulation; however, these changes were not significant. Notably, *pbx4* and *ezh2* are key regulators of developmental gene expression and chromatin remodeling, likely contributing to the enhanced proliferative capacity observed in cardiomyocytes. Additionally, the upregulation of *acss3*, a metabolic regulator, and *thbs3a*, a crucial factor in extracellular matrix remodeling, highlights the potential role of ATF3 in promoting structural changes and fibrosis in the myocardium. These results suggest that ATF3 overexpression drives a transcriptional shift favoring enhanced cell growth and developmental activity while suppressing apoptosis. This reprogramming likely contributes to the structural and functional cardiac abnormalities observed in *Tg(myl7:ATF3)* zebrafish, including hypertrophic remodeling and fibrosis. Consistent with these transcriptomic findings, the TUNEL assay revealed a marked reduction in TUNEL-positive signals in Tg(*myl7:ATF3*) hearts compared with WT controls (Supplementary Fig. S6), indicating that ATF3 overexpression is associated with reduced apoptotic cell death in the adult zebrafish heart.

## Discussion

The findings of this study indicate that ATF3 overexpression in cardiomyocytes is associated with cardiac hypertrophy. Transgenic zebrafish overexpressing ATF3 exhibited numerous structural and functional abnormalities resembling an HCM-like phenotype. In addition to inflammation, fibrosis and macrophage infiltration were observed alongside the HCM-like phenotype. The trabecular myocardium, which constitutes the majority of the ventricular wall among the three layers in zebrafish—trabecular, primordial, and cortical myocardium—was significantly thickened^16^. Additionally, the structure of the endocardial layer overlying the trabecular myocardium was disrupted. At the cellular level, cardiomyocyte hypertrophy was evident in the hearts of zebrafish overexpressing ATF3, specifically Tg(*myl7:ATF3*) zebrafish, characterized by an increase in cardiomyocyte size and cell proliferation.

Previous studies have consistently reported an association between ATF3 and cell proliferation. For instance, ATF3 promotes adult T-cell leukemia cell proliferation by upregulating CDC2 and cyclin E2^17^. In keloid fibroblast cells, increased ATF3 expression enhances the levels of tumor growth factor-beta (TGF-β) receptor RI and RII and promotes the phosphorylation of Smad2 and Smad3. This process regulates the proliferation and collagen production of keloid fibroblasts through the TGF-β/Smad pathway^18^. Moreover, during osteoclastogenesis, ATF3 deficiency inhibits RANKL-induced cell proliferation and modulates the proliferation of osteoclast precursors by regulating cyclin D1 expression^19^. Consistent with these findings, our zebrafish model exhibited increased proliferation of cardiac cells, including cardiomyocytes, in response to ATF3 overexpression. This finding suggests that ATF3 is associated with increased cell proliferation in the heart, potentially through mechanisms similar to those reported in other cell types.

Previous studies in mammalian models have linked ATF3 to fibroblast activation and macrophage proliferation in the injured heart, supporting its general role in modulating cellular proliferation across multiple cardiac cell types^20,21^. This study confirms that ATF3 promotes cardiomyocyte proliferation when specifically overexpressed in these cells. Although a subset of PH3-positive proliferating cells co-localized with cardiomyocytes, others did not. This result suggests that additional cardiac cell types also undergo proliferation in response to ATF3 overexpression. However, the identity and functional role of these non-cardiomyocyte proliferating cells remain unclear and warrant further investigation.

Quantitative sequencing revealed transcriptomic changes suggesting a potential role for ATF3 in regulating apoptosis, synaptic function, and developmental pathways in cardiomyocytes. The downregulation of *Syngap1b* and *dmrt2a* indicates impaired synaptic signaling, whereas the suppression of *dedd* and *buc2l* reflects alterations in apoptotic regulation. Conversely, the upregulation of *pbx4* and *ezh2*—key regulators of developmental processes—along with *acss3* and *thbs3a*, which are involved in metabolic activity and extracellular matrix remodeling, suggests a transition toward a more proliferative and developmental state. These findings imply that ATF3 overexpression is linked to transcriptional reprogramming associated with cardiac remodeling, potentially contributing to the structural and functional abnormalities observed in Tg(*myl7:ATF3*) zebrafish.

Collectively, these observations support a working model in which ATF3-driven transcriptional reprogramming activates developmental and proliferative gene networks while suppressing apoptotic signaling. This dual regulation may shift cardiomyocytes toward a growth-permissive and metabolically active state, leading to hypertrophic remodeling. Additionally, the downregulation of the ion channel gene *kcnq1.1*, along with the prolonged QT interval observed in ECG recordings, suggests that ATF3 overexpression may indirectly alter cardiac electrical properties as a secondary effect of structural and transcriptional remodeling of the myocardium.

ECG measurements of Tg(*myl7:ATF3*) zebrafish hearts revealed a phenotype resembling LQTS. LQTS is a cardiac disorder characterized by a prolonged QT interval on ECG, which increases the risk of ventricular arrhythmias and sudden cardiac death. This syndrome can be either congenital or acquired, with various genetic mutations or medications contributing to its development. LQTS primarily results from functional abnormalities in ion channels within myocardial cells and is closely associated with genetic defects in ion channel-related genes, such as *KCNQ1*, *KCNH2*, and *SCN5A*^22^. However, no studies have explored the relationship between ATF3 and LQTS, nor has it been associated with genes implicated in ion channel function. Although an LQTS-like phenotype has been observed in zebrafish overexpressing ATF3, further research is warranted to determine whether ATF3 directly affects ion channels to cause this phenotype or if it reflects symptoms arising from cardiac dysfunction associated with the HCM-like phenotype.

Our data reveal concurrent increases in cardiomyocyte proliferation and structural remodeling alongside prolonged QT intervals; however, the direct mechanistic relationship between these phenomena remains unclear. The reduced *kcnq1.1* expression observed in Tg(*myl7:ATF3*) hearts suggests that ATF3 overexpression may partly influence cardiac electrophysiology through altered ion channel gene regulation, possibly as a secondary effect of hypertrophic remodeling and fibrosis. Further functional experiments are required to establish a causal link between ATF3-induced proliferation and electrical dysfunction.

In conclusion, the results of this study indicate that the ectopic expression of human ATF3 is associated with cardiac abnormalities and dysfunction. Overexpression of ATF3 increases cardiomyocyte size and proliferation, induces macrophage infiltration, and is associated with fibrosis and alterations in myofibril structure. Zebrafish ECG analysis revealed a prolonged QT interval, resembling an LQTS-like phenotype, ultimately leading to HCM. RNA sequencing further revealed altered expression of genes associated with cell proliferation. These findings suggest that ATF3, rather than playing a protective role in the zebrafish heart, is associated with cardiac hypertrophy. Although our results demonstrate concurrent increases in cardiomyocyte proliferation and hypertrophic remodeling, these findings reflect correlation rather than proven causation. Further research is necessary to determine whether ATF3 induces hypertrophy through direct regulation of genes involved in cell growth and proliferation. The specific mechanisms by which ATF3 regulates cardiomyocyte proliferation should also be determined in future studies.

## Methods

### Zebrafish maintenance

All procedures used in this study were approved by the Institutional Animal Care and Use Committee (IACUC) of the Korea Disease Control and Prevention Agency (Approval No. KDCA-IACUC-24–034) and were performed in accordance with the relevant guidelines and regulations. This study was conducted following the ARRIVE guidelines. The transgenic zebrafish lines included Tg(*myl7:ATF3*), *Tg(myl7:GFP)*^23^, Tg(*fli1a:GFP*) (also described as Tg(*fli1:EGFP*) in other publications)^24^, and Tg(*mpeg1:gal4vp16;uas:EGFP*)^25^. All zebrafish were maintained in a recirculating water system at 28 ± 0.5 °C under a 14/10 h light–dark cycle. The fish were fed a combination of dry food (Gemma Micro 75 or 300, Skretting, Stavanger, Norway) and live brine shrimp twice daily. Developmental stages were determined based on dpf and mpf^26^. Adult zebrafish (approximately 4–12 mpf, male and female) were used in this study.

### Generation of Tg(myl7:ATF3) zebrafish

A 903 bp fragment of the zebrafish *myl7* (also known as *cmlc2*) promoter was subcloned into the pCS2^+^ vector upstream of the human *ATF3* gene to generate the Tg(*myl7:ATF3*) transgenic zebrafish line. The resulting construct was linearized and microinjected into one-cell-stage zebrafish embryos. The injected embryos were raised to adulthood and outcrossed with WT zebrafish. Genomic DNA was extracted from F1 embryos for genotyping to identify transgenic founders. Confirmed founders (F0) were then outcrossed to WT zebrafish to establish stable transgenic lines.

### Whole-mount in situ RNA hybridization

Whole-mount in situ RNA hybridization was performed as previously described^27^. The coding sequence of human *ATF3* (NM_001030287.3) was cloned into the pGEM-T Easy vector (Promega, Madison, WI, USA) and used as a template to synthesize antisense RNA probes. The probe was synthesized from a 546 bp fragment corresponding to nucleotides 82–627 of the human ATF3 transcript, encompassing the complete coding sequence from the ATG start codon to the stop codon. Digoxigenin (DIG)-labeled antisense RNA probes were transcribed in vitro using T7 RNA polymerase (Roche, Basel, Switzerland) and purified by lithium chloride precipitation. Zebrafish embryos were fixed in 4% paraformaldehyde (PFA) overnight at 4 °C, dehydrated, and stored in 100% methanol at − 20 °C. After rehydration, the embryos were treated with proteinase K (approximately 10–20 µg/mL) at room temperature (25 °C) for permeabilization, post-fixed in 4% PFA, and hybridized with DIG-labeled RNA probes at 63 °C overnight. Following hybridization, the embryos were washed with SSC buffers, blocked with 2% normal sheep serum and bovine serum albumin (BSA) in phosphate-buffered saline with 0.1% Tween-20 (PBST), and incubated with anti-DIG-AP Fab fragments (Roche) at 4 °C overnight. Signal detection was performed using NBT/BCIP (Roche) as the chromogenic substrate. Stained embryos were imaged using an upright microscope (Axio Imager, Carl Zeiss, Oberkochen, Germany).

### Cryosection of zebrafish hearts

Zebrafish hearts were harvested and fixed in 4% PFA overnight at 4 °C. After fixation, the hearts were washed in PBST and cryoprotected by incubation in a 30% sucrose solution overnight at 4 °C. The tissues were then embedded in optimal cutting temperature compound (Tissue-Tek, Sakura, Torrance, CA, USA) and cryosectioned using a cryostat (CM3050S, Leica). Sections were cut to a thickness of 5 μm for hematoxylin and eosin (H&E) and trichrome staining, and 10–12 μm for immunofluorescence and TUNEL staining.

### H&E and trichrome staining

For H&E staining, the sections were first stained with hematoxylin for 20 s, followed by eosin for an additional 20 s. For trichrome staining, the sections were stained using the Trichrome Stain Kit (ab150686, Abcam, Cambridge, UK) according to the manufacturer’s instructions. After staining, the sections were mounted with a mounting medium (Surgipath® MM 24 Mounting Media, Leica), and images were captured using an inverted microscope (EVOS M5000, Thermo Fisher Scientific, Waltham, MA, USA).

### Immunofluorescence and TUNEL staining

For immunofluorescence staining, the sections were blocked with 2% normal sheep serum and BSA in phosphate-buffered saline (PBS) for 1 h at room temperature (25 °C). The sections were then incubated overnight at 4 °C with primary antibodies. After washing with PBS, the sections were incubated overnight at 4 °C with secondary antibodies. The slides were mounted with Fluoroshield (F6182, Sigma-Aldrich, St. Louis, MO, USA). The following antibodies were used: PCNA (P8825, Sigma-Aldrich; 1:200), Phospho-Histone H3 (Ser10) (06–570, Millipore; 1:100), ATF3 (HPA001562, Atlas Antibodies, Stockholm, Sweden; 1:200), GFP (ab13970, Abcam; 1:200), Alcama (GTX128399, GeneTex, Irvine, CA, USA; 1:200), Laminin (L9393, Sigma-Aldrich; 1:30), Goat anti-Rabbit IgG, Alexa Fluor™ 488/568 (A-11008/A-11011, Invitrogen, Waltham, MA, USA; 1:1,000), Goat anti-Mouse IgG, Alexa Fluor™ 488/568 (A-11001/A-11004, Invitrogen; 1:1,000), Goat anti-Chick IgY H&L, Alexa Fluor™ 488 (ab150169, Abcam; 1:1,000), and DAPI (62,248, Thermo Fisher Scientific; 1:1,000). TUNEL staining was performed using an In Situ Cell Death Detection Kit (12,156,792,910, Roche) according to the manufacturer’s instructions.

### Imaging and statistical analyses

Heart morphology images of adult zebrafish were captured using a stereomicroscope (MZ 8, Leica), and the ventricular area was quantified from these images using Fiji (ImageJ) after scale calibration. The ventricular boundary was manually outlined with the freehand selection tool to obtain the projected area (mm^2^). Imaging for fluorescence microscopy was performed using a confocal microscope (FV3000, Olympus, Tokyo, Japan), and images were analyzed with cellSens software (version 4.1, Olympus) to process and quantify the data. For TEM, images were captured with a transmission electron microscope (DE/LIBRA 120, Carl Zeiss, Oberkochen, Germany) and analyzed with Fiji (ImageJ) for quantitative measurements such as Z-line width. Statistical analysis was performed using GraphPad Prism 10.

### ECG analysis

Adult WT and Tg(*myl7:ATF3*) zebrafish were anesthetized using a 0.02% tricaine solution until they reached anesthesia stage 3. The zebrafish were positioned on a damp sponge with their ventral sides facing upward. For ECG recording, two copper-core electrodes were invasively inserted into the chest muscle layer to a depth of approximately 0.5 mm. All experiments were conducted at room temperature (25 °C). ECG data were recorded inside a steel-shielded chamber designed to block electromagnetic interference and sound, minimizing external noise (SonTek, San Diego, CA, USA). A 0.5–55 Hz bandpass filter (Biopac software, Goleta, CA, USA) was applied to eliminate electrical noise at 60 Hz. ECG signals were recorded at a sampling rate of 10 kSa/s, and the data were saved on a laptop using Biopac Student Lab 4.1. A complete heartbeat cycle featuring distinct P, R, and T waves was manually identified. Analysis was performed on regions where uniform waveforms were repeated more than five times. ECG intervals, including the PP and QT intervals, were manually measured based on these uniform waveforms. The heart rate (bpm) was calculated using the formula: 60 divided by the PP interval (in seconds). The PP interval represents the time between two consecutive P waves, corresponding to atrial depolarization in the ECG signal. The QT interval was defined as the duration from the upstroke of the R wave to the end of the T wave, marking repolarization termination. QT intervals were then normalized to heart rate using Bazett’s formula: the corrected QT interval was calculated by dividing the QT interval by the square root of the PP interval. ECG data were recorded as described above and analyzed by Zefit Inc. (Daegu, Republic of Korea) in a blinded manner. The analysis files were anonymized (e.g., “Group 1–Fish 1”) without genotype information, and the genotype key was provided only after the completion of data analysis.

### Quant-RNA Seq analysis

Raw Quant-seq reads were quality-filtered and trimmed to eliminate adapter sequences and low-quality bases using Trim Galore (version 0.6.6). The processed reads were aligned to the GRCz11.109 reference genome using STAR (version 2.7.11a). Transcript assembly, abundance estimation, and differential gene expression analysis were performed using RSEM (version 1.3.3). Feature counting was conducted to obtain FPKM values, and differential expression analysis was performed using DESeq2 (version 1.32.0). Graphical representations of the Quant-seq results were generated using Python (version 3.8.12), matplotlib (version 3.5.1), and seaborn (version 0.11.2).

### Quantitative real-time PCR

Total RNA was extracted from zebrafish larvae or adult hearts using TRIzol reagent (15596018, Invitrogen), and cDNA was synthesized using the RNA to cDNA EcoDry™ Premix (Oligo dT) (639543, TaKaRa, Shiga, Japan) according to the manufacturer’s instructions. Quantitative real-time PCR (qRT-PCR) was performed using Power SYBR Green PCR Master Mix (4367659, Applied Biosystems, Waltham, MA, USA) on a QuantStudio™ 6 Flex Real-Time PCR System (96-well format, Applied Biosystems). Gene expression levels were normalized to β-actin as an internal control, and relative expression was calculated using the 2^–ΔΔCt^ method. Primer sequences used for qRT-PCR are listed in Supplementary Table 3.

### Sequence alignment of ATF3 orthologs

Amino acid sequences of ATF3 orthologs were retrieved from Ensembl for *H. sapiens* (ENSP00000344352), *M. musculus* (ENSMUSP00000027941), and *D. rerio* (ENSDARP00000027550). Multiple sequence alignment was performed using Clustal Omega (EMBL-EBI) with default parameters, and the alignment was visualized in Jalview (v2.11.5.0) using the Clustal color scheme. Percentage ID values were calculated using the pairwise alignment feature of Jalview.

## Supplementary Information

Supplementary Information 1.

Supplementary Information 2.

Supplementary Information 3.

Supplementary Information 4.

## Data Availability

Quant-RNA seq datasets generated and analyzed during the current study are available in the Gene Expression Omnibus repository, GSE295384 and can be accessed at https://www.ncbi.nlm.nih.gov/geo/query/acc.cgi?acc=GSE295384.
