# Supplementary material for: ATF3 overexpression is associated with cardiac hypertrophy and electrical dysfunction accompanied by enhanced cardiac cell proliferation in zebrafish
Source: Sci Rep. 2025 Dec 26;16:3143. doi: 10.1038/s41598-025-33025-3 (PMC12830903; doi:10.1038/s41598-025-33025-3)
Supplement: Supplementary file 4 — Supplementary Information 4. [file 41598_2025_33025_MOESM4_ESM.pdf]

ATF3 Overexpression is Associated with Cardiac Hypertrophy and Electrical Dysfunction Accompanied by Enhanced Cardiac Cell Proliferation in Zebrafish

Eunmi Kim, Jinho Kim, Hyun-Yi Moon, Ji Yeon Kim, Myong-Ho Jeong, Geun-Young Kim, Seung Hee Lee, Chul-Hong Kim, Jung-Woong Kim, Won-Ho Kim

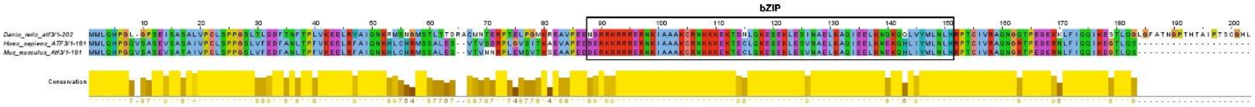

Supplementary Figure S1. Sequence alignment of ATF3 orthologs

Amino acid sequence alignment of ATF3 proteins from *Homo sapiens* (ENSP00000344352), *Mus musculus* (ENSMUSP00000027941), and *Danio rerio* (ENSDARP00000027550), generated using Clustal Omega (EMBL-EBI) and visualized in Jalview (v2.11.5.0). Conserved residues are indicated in color, and the basic leucine zipper (bZIP) domain (box; approximately residues 89–151 in human ATF3) is evident across species. Percentage ID (calculated in Jalview) was 95% between human and mouse and 71% between human and zebrafish.

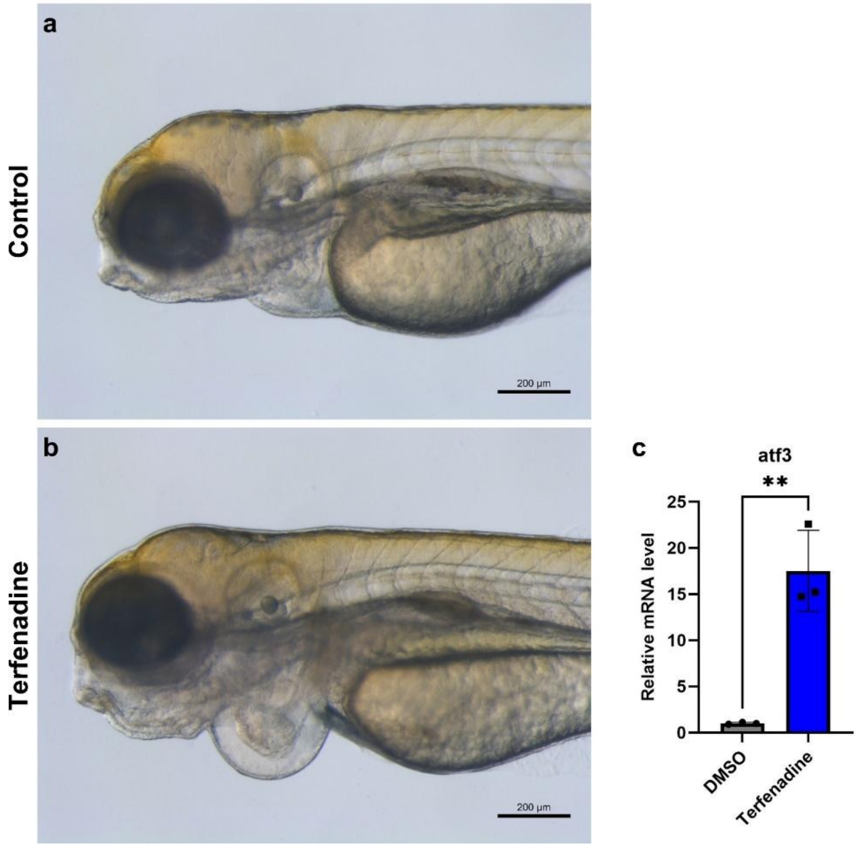

Supplementary Figure S2. Terfenadine-induced cardiac stress increases endogenous *atf3* expression in zebrafish larvae

(**a, b**) Representative bright-field images show cardiac enlargement and pericardial edema in terfenadine-treated larvae compared with DMSO controls at 4 dpf. (**c**) Quantitative PCR analysis demonstrates a significant increase in *atf3* mRNA levels in terfenadine-treated zebrafish relative to controls. Data are presented as the mean ± SD (n = 3 pooled biological replicates per group, each composed of 40 larvae) and analyzed using an unpaired two-tailed t-test; **P < 0.01.

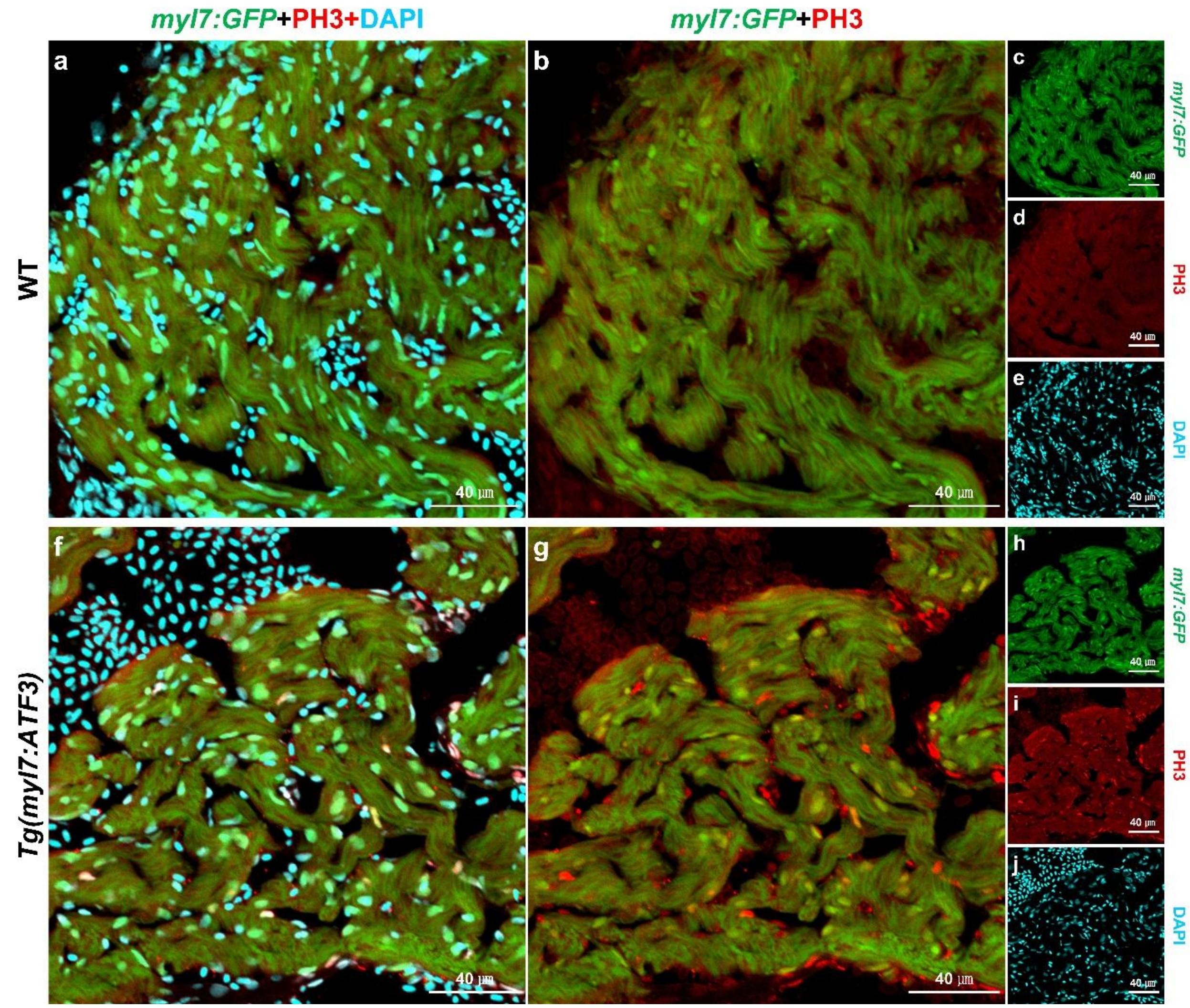

Supplementary Figure S3. Increased proliferating cells in *Tg(myl7:ATF3);myl7:GFP* zebrafish hearts revealed by PH3 and *myl7:GFP* co-staining

(**a, b**) Confocal images of adult control zebrafish hearts (*Tg(myl7:GFP)*) show cardiomyocytes labeled with *myl7:GFP* (green), proliferating cells labeled with PH3 (red), and nuclei counterstained with DAPI (cyan). (**a**) Merged image of *myl7:GFP*, PH3, and DAPI; (b) *myl7:GFP* and PH3 two-channel merge. (**c–e**) Single-channel images from the same field in (**a**): *myl7:GFP* (**c**), PH3 (**d**), and DAPI (**e**). (**f, g**) Corresponding merged images in *Tg(myl7:ATF3);myl7:GFP* hearts. (**f**) Merged *myl7:GFP*/PH3/DAPI; (**g**) *myl7:GFP*/PH3 two-channel merge. (**h–j**) Single-channel images from the same field in (**f**): *myl7:GFP* (**h**), PH3 (**i**), and DAPI (**j**). *Tg(myl7:ATF3)* hearts show increased $PH3^{+}$ nuclei compared with the WT.

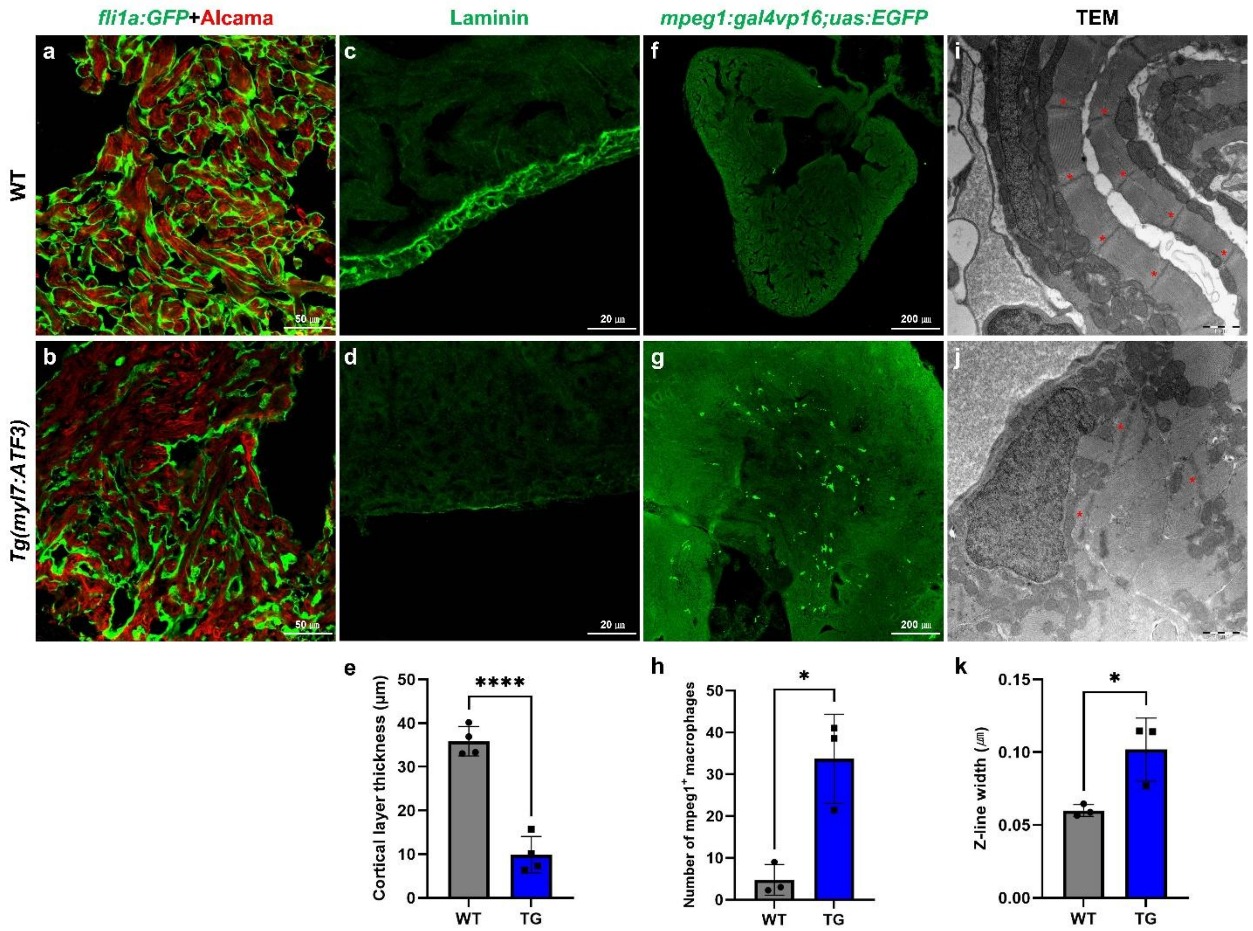

Supplementary Figure S4. Comparison of myocardium and cardiomyocyte structures and inflammation in WT and Tg(*myl7:ATF3*) zebrafish

(**a, b**) Alcama antibody staining reveals abnormal endocardial structure in Tg(*fli1a:GFP*) and Tg(*myl7:ATF3*);Tg(*fli1aGFP*) zebrafish at 4 mpf (n = 3 hearts per group). (**c, d**) Laminin antibody staining demonstrates damage to the cortical myocardium in Tg(*myl7:ATF3*) zebrafish at 12 mpf. (e) Quantification of Laminin^{+} cortical myocardium thickness (n = 4 hearts per group) (**f, g**) Tg(*mpeg1:gal4vp16;uas:EGFP*) staining shows increased macrophage infiltration in Tg*(myl7:ATF3*) zebrafish hearts at 12 mpf. Quantification of macrophage infiltration within the ventricular region is shown in (**h**) (n = 3 hearts per group). (**i, j**) Transmission electron microscopy images reveal disrupted myofibril structure in Tg(*myl7:ATF3*) zebrafish cardiomyocytes at 12 mpf. Quantification of z-line width (asterisk) of sarcomeres is shown in (**k**) (n = 3 hearts per group). Data are presented as the mean ± SD and are analyzed using an unpaired two-tailed t-test. *P < 0.05, ****P < 0.0001. mpf, months post-fertilization

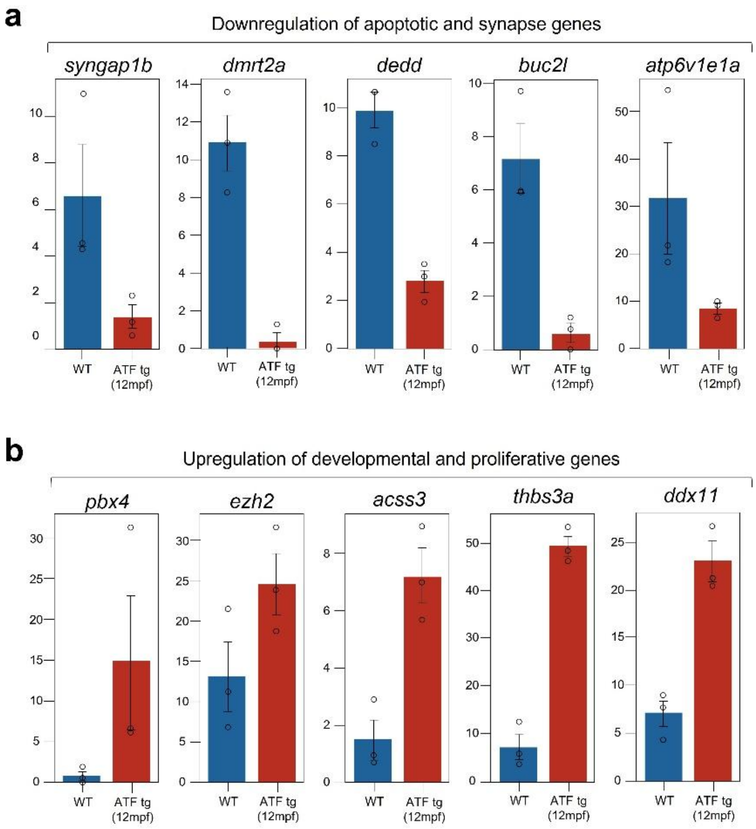

Supplementary Figure S5. Expression levels of selected DEGs in adult zebrafish hearts

(**a, b**) Expression levels of selected DEGs in the 12 mpf group. (**a**) Apoptotic and synapse-related genes (*syngap1b, dmrt2a, dedd, buc2l,* and *atp6v1e1a*). (**b**) Developmental and proliferative genes (*pbx4, ezh2, acss3, thbs3a,* and *ddx11d*). Each dot represents an individual sample, and error bars indicate the SD. DEG, differentially expressed gene

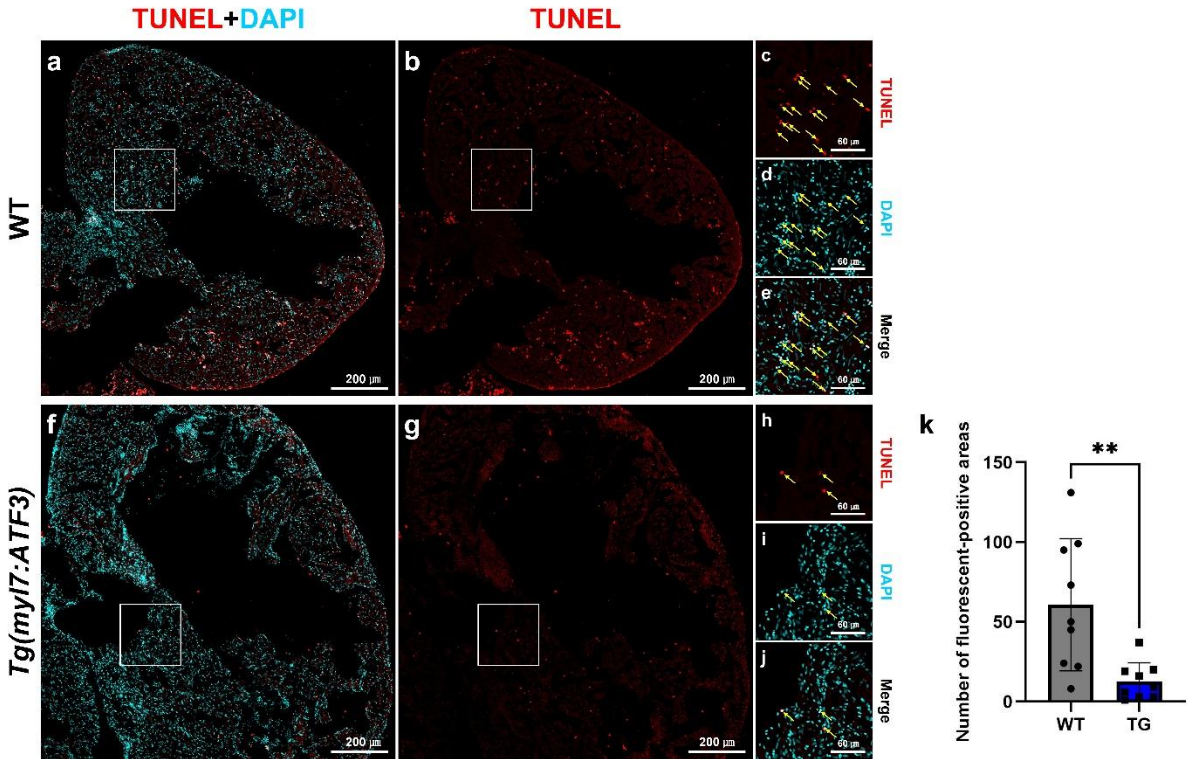

Supplementary Figure S6. Apoptotic cell signals in Tg(*myl7:ATF3*) hearts revealed by the TUNEL assay

Representative fluorescence images of TUNEL staining in adult zebrafish hearts from WT (a–e) and Tg(*myl7:ATF3*) (**f**–**j**). (**a, b, f, g**) DAPI + TUNEL and TUNEL-only images at low magnification. (**c**–**e**, **h**–**j**) display high-magnification views corresponding to the boxed regions in (**a, b**) and (**f, g**), respectively, showing DAPI, TUNEL, and merged channels. (**k**) Quantification of TUNEL-positive area per ×100 field (based on representative images in **a, b, f, g**). Data are presented as the mean ± SD (n = 3 hearts per group; three ventricular sections analyzed per heart). Statistical analysis was performed using an unpaired two-tailed t-test; **P < 0.01.
